# Anticipation of novel environments enhances memory for incidental information

**DOI:** 10.1101/lm.053392.121

**Published:** 2021-08

**Authors:** Danlu Cen, Christos Gkoumas, Matthias J. Gruber

**Affiliations:** Cardiff University Brain Research Imaging Centre (CUBRIC), School of Psychology, Cardiff University, Wales CF24 4HQ, United Kingdom

## Abstract

Novelty is a potent driver of learning, but little is known about whether anticipation of novelty can enhance memory for incidental information. Here, participants incidentally encountered objects while they actively navigated toward novel or previously familiarized virtual rooms. Across immediate and delayed surprise memory tests, participants showed superior recollection for incidental objects encountered while anticipating novel as compared with familiarized rooms. Furthermore, memory for incidental objects correlated positively with between-participants average curiosity about novel rooms but negatively with within-participants trial-specific curiosity. Our findings contribute to the growing literature on how salient processes impact memory for incidental material.

Accumulating evidence suggests that being in a state of high motivation influences the likelihood of memory formation and later consolidation. For instance, anticipation of extrinsic reward such as monetary gain improves memory of incentivized information (e.g., Adcock et al. 2006; Wolosin et al. 2012; Gruber et al. 2013), and even of incidental, neutral information that is merely encountered in temporal proximity to the reward (Mather and Schoeke 2011; Stanek et al. 2019), supported by the dopaminergic system and memory-related regions (Wittmann et al. 2005; Adcock et al. 2006; Wolosin et al. 2012; Gruber et al. 2016; Murty et al. 2017).

Like reward, novelty is also a potent motivational signal. Theoretical accounts propose that novelty has intrinsic reward values and is a driving force motivating exploration of novel environments for potential sources of rewards (Kakade and Dayan 2002; Düzel et al. 2010). In support of this view, converging evidence from animals (Ljungberg et al. 1992; Wang et al. 2010; Redondo and Morris 2011) and humans (Schott et al. 2004; Bunzeck and Düzel 2006; Fenker et al. 2008; Bunzeck et al. 2014; Murty and Adcock 2014; Herweg et al. 2017) has revealed that the dopaminergic system responds to novel stimuli in the absence of immediate reward reinforcement. Consistent with reward anticipation, cues that predict upcoming novel stimuli are accompanied by increased recruitment of dopaminergic circuit and memory-related regions during the anticipation of novel information (Wittmann et al. 2007). These findings implicate that anticipation of novelty could induce a high motivational state and lead to enhanced memory for incidental information learned during such a state. Surprisingly, this behavioral possibility has so far not been tested.

A separate, fledgling field of research has shown that states of curiosity—the intrinsic motivation to acquire novel information (Berlyne 1960; Loewenstein 1994; Litman et al. 2005)—also recruits activity within the dopaminergic circuit (for a review, see Gruber et al. 2019). Specifically, studies showed that states of curiosity enhance memory for incidental information that is encountered during states of high compared with low curiosity (Gruber et al. 2014; Galli et al. 2018; Stare et al. 2018; Fandakova and Gruber 2021; Murphy et al. 2021). However, it is not known whether the positive effects of novelty and curiosity on incidental memory actually reflect the same phenomenon (i.e., all novel information equally enhances memory, but curiosity would not further modulate memory), or whether they rely on partly distinct processes (i.e., the level of curiosity about novel information additionally affects memory).

Here, we addressed the question of whether anticipation of novelty enhances memory for incidental information. We developed a novel virtual reality (VR) paradigm that allows participants to actively navigate along a zigzag-shaped pathway toward novel or previously familiarized rooms (see Fig. 1A,B). At the beginning of a trial, participants were cued with a room label that indicated the novelty or familiarity level of the room. On the pathway, participants encountered six daily life neutral objects separately allocated at the corners of the pathway (see Fig. 1C). To make the encoding of these objects as incidental as possible, no explicit task was administrated on these objects. To dissociate between potential memory enhancements related to encoding or memory consolidation mechanisms (Bunzeck et al. 2010; Murayama and Kitagami 2013; Gruber et al. 2014; Javadi et al. 2015; Stare et al. 2018), participants were given a surprise memory test for the objects that they had seen on the pathway leading to the rooms, either immediately or 24 h after the encoding phase (see Fig. 1D). The 36 objects that had been seen in the encoding phase (i.e., the six objects on the pathway leading up to the six different rooms) and 18 lures were presented on the screen one at a time in a randomized and intermixed sequence. For each presented object, participants indicated whether it was “remembered,” “familiar,” or “new.” We predicted that later recollection should be enhanced for the objects leading to the novel compared with familiarized rooms.

**Figure 1.**
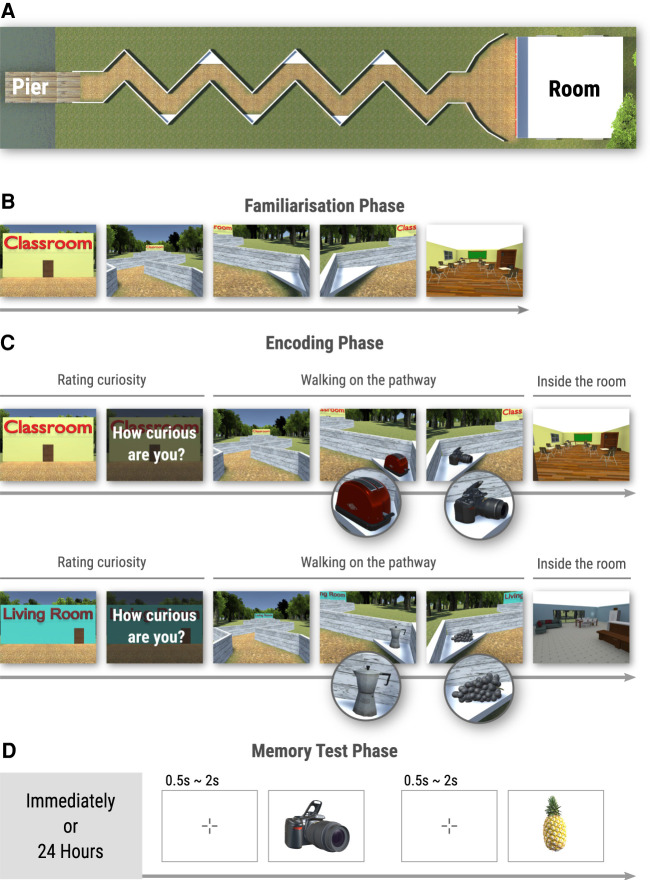
Virtual environment and experimental design. (*A*) The virtual environment contained a pier (the starting point for each trial), a zigzag-shaped pathway, and a room. The experiment consisted of three phases: familiarization (*B*), encoding (*C*), and memory test (*D*). During the familiarization phase, the participants explored three rooms (for example, Bedroom, Classroom, and Gym). In a familiarization trial, participants were first informed about the room they were going to visit (e.g., Classroom), and then they walked along the pathway and entered the room. (*C*) During the encoding phase, participants visited all six rooms, including the three visited in the familiarization phase (familiarized condition) and three novel rooms (novel condition). Two example trials are illustrated: one in the familiarized condition (e.g., Classroom visited during the familiarization phase) and one in the novel condition (e.g., Living Room that was not previously visited). Participants started at the pier with the type of to-be-visited room clearly visible (e.g., Classroom or Living Room) and rated their curiosity on a Likert scale from 1 (“not at all curious”) to 7 (“extremely curious”). Then, participants navigated to the room through a zigzag-shaped pathway. On the pathway to the room, they would see six objects, each at the corner of the pathway. For example, a toaster and a camera on the pathway in the familiarized condition (*top* panel), and a moka pot and grapes on the pathway in the novel condition (*bottom* panel). (*D*) Either immediately following the encoding phase (the immediate memory test group) or after 24 h (the delayed memory test group), participants took part in a surprise recognition memory test for objects encountered on the pathway leading to the rooms. In each trial, they were shown an object, which could be an object seen on the pathway during the encoding phase (e.g., camera), or a new object as a lure (e.g., pineapple). Participants were instructed to indicate whether the object was “remembered,” “familiar,” or “new.”

In addition, ahead of navigating through the pathway in each trial, participants rated their curiosity for the room on a Likert scale from 1 (“not at all curious”) to 7 (“extremely curious”) (see Fig. 1C). Based on prior findings on perceptual curiosity (i.e., curiosity for novel and uncertain stimuli) (Jepma et al. 2012), we expected novel rooms to elicit higher curiosity as compared with familiarized rooms. Importantly, the curiosity ratings allowed us to explore whether curiosity additionally strengthened the potential memory effects of novelty anticipation on incidental information or whether curiosity is simply a by-product of novelty-related memory enhancements and therefore does not lead to any additional effects on memory beyond the effects of novelty.

We recruited 82 participants. All reported normal hearing, normal or corrected to normal vision and were naïve about the purpose of the study. Half of them were assigned to the condition that involved an immediate memory test, and the other half to the condition that involved a 24-h delayed memory test. Three participants were excluded for having an overall average hit rate of “remember” responses for old objects lower than the false alarm rate (*N* = 2) or for having curiosity ratings <3 SDs from group mean (*N* = 1). As a result, a total of 79 participants (14 males; mean ± SD = 21.38 ± 3.86 yr of age) were included in the final analysis. Additional methodological details are included in the Supplemental Material.

Recollection performance was examined by response accuracy, which was calculated as the corrected hit rate for old objects (i.e., proportion of correct “remembered” responses for old objects—proportion of incorrect “remembered” responses for new objects). Here we focused on recollection performance because it is thought to be dependent on the hippocampus (Yonelinas 2002), and given theoretical models and findings on reward, novelty and curiosity, recollection would be expected to show the strongest effects related to novelty and curiosity (Lisman and Grace 2005; Wittmann et al. 2007; Düzel et al. 2010; Shohamy and Adcock 2010; Gruber et al. 2016; Murphy et al. 2021). Data analysis details are included in the Supplemental Material. In the present study, all tests reported are two-tailed. Data and analysis scripts can be downloaded from https://osf.io/fbkxj.

As illustrated in Figure 2, recollection accuracy was better for the immediate (*M* = 32.29%, 95% confidence interval [CI] = [28.08%, 36.51%]) compared with the delayed condition (*M* = 21.25%, 95% CI = [17.52%, 24.98%]). More importantly, recollection accuracy was better for the objects leading to the novel rooms (*M* = 28.95%, 95% CI = [24.69%, 33.2%]) than for those leading to the familiarized rooms (*M* = 24.73%, 95% CI = [20.69%, 28.78%]). A 2 × 2 mixed ANOVA on recollection accuracy with memory test (immediate vs. delayed) as a between-subject factor and room novelty (novel vs. familiar) as a within-subject factor revealed a significant main effect of memory test (*F*_(1,77)_ = 9.20, *P* < 0.01, *η*_p_^2^= 0.11) and room novelty (*F*_(1,77)_ = 6.39, *P* = 0.014, *η*_*p*_^2^ = 0.077), without any significant interaction (*F*_(1,77)_ = 0.079, *P* = 0.78), suggesting that novelty anticipation enhanced incidental recollection and did not differ between immediate and delayed memory. This novelty-related memory enhancement was most prominent for the objects closest to the starting point (intercept β = 8.56, *P* < 0.01), and tended to decrease over distance as participants approached the rooms (slope β = −1.69, *P* = 0.069) (see Supplemental Fig. S1). For familiarity-based recognition accuracy, we did not find a significant main effect or interaction with room novelty (all *P*s > 0.05) (see Supplemental Fig. S3; Supplemental Table S1), suggesting that the beneficial effect of novelty anticipation is specific to recollection.

**Figure 2.**
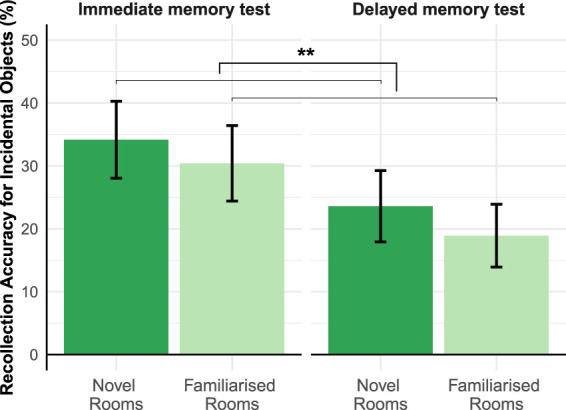
Mean recollection accuracy (=“remember” responses for old objects − “remember” responses for new objects) for objects on the pathway leading toward the rooms. The data are averaged across the participants for the immediate (*N* = 40) and delayed (*N* = 39) memory tests separately. Dark-green bars show the mean recollection accuracy for the objects leading to the novel rooms, and light-green bars for objects leading to the familiarized rooms. Error bars depict 95% confidence interval. The mean ([95% CI]) for the four conditions are Immediate–Novel (34.17% [28.04%, 40.29%]), Immediate–Familiarized (30.42% [24.41%, 36.42%]), Delayed–Novel (23.30% [17.74%, 28.85%]), and Delayed–Familiarized (18.58% [13.67%, 23.50%]). Asterisks (**) indicate the difference between novel and familiarized conditions at the *P* < 0.01 significance level.

As expected, participants reported to be more curious toward novel (*M* = 5.76, 95% CI = [5.59, 5.92]) compared with familiarized rooms (*M* = 2.77, 95% CI = [2.52, 3.02]; *t*_78_ = −19.42, *P* < 0.001) (see Supplemental Fig. S2). Next, we explored the effect of curiosity on recollection by looking at the relationship between recollection accuracy for incidental objects on the pathway leading to novel rooms and curiosity ratings for the novel rooms at both interindividual and intraindividual levels. We performed a multilevel analysis with participant as the random effect, individual curiosity ratings for each of the three novel rooms as the level 1 predictor, and average curiosity rating across the three novel rooms and memory test (immediate and delayed) as the level 2 predictors (see the Supplemental Material for more details). At the interindividual level, we found a significant positive relationship between average curiosity rating and recollection accuracy (*b* = 6.34, *P* = 0.027) (see Fig. 3A), indicating that recollection accuracy increased as participants were generally more curious about novel rooms. In contrast, however, at the intraindividual level, the model revealed a significant negative relationship between curiosity rating per novel room and recollection accuracy (*b* = −6.69, *P* < 0.001) (see Fig. 3B), indicating that at the intraindividual level, recollection of incidental objects decreased with higher curiosity for a particular novel room (see below for further discussion of the opposing interindividual and intraindividual effects of curiosity on memory).

**Figure 3.**
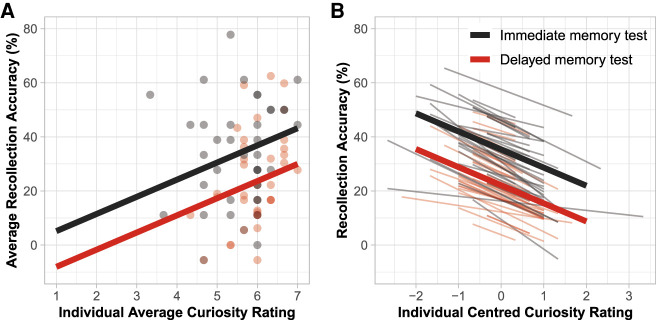
Results of the multilevel analysis examining the relationship between curiosity for novel rooms and recollection accuracy of incidental objects on the pathway leading to the novel rooms at the interindividual level (*A*) and the intraindividual level (*B*). In *A*, solid lines illustrate the fixed effect of average curiosity rating across three novel rooms separately for the immediate (black) and delayed (red) memory tests. Each dot represents data from a participant. In *B*, thick solid lines illustrate the fixed effect of curiosity rating per novel room, separately for the immediate (black) and delayed (red) memory tests. Thin solid lines represent random effects for individual participants; that is, each thin line shows the predicted relationship between curiosity and recollection accuracy for an individual participant.

Taken together, the present study demonstrated that anticipation of novelty promotes memory for incidental objects. Specifically, anticipation of novelty significantly improved recollection but not familiarity-based recognition, suggesting that this novelty-related effect is specific to hippocampus-dependent memory. Our findings on novelty and incidental recollection are consistent with prior studies in animals that have shown enhanced memory for unrelated information learned before and after novelty exposure (e.g., Li et al. 2003; Wang et al. 2010). In humans, studies consistently demonstrated beneficial effects on memory for unrelated information learned after novelty exposure (Fenker et al. 2008; Schomaker et al. 2014; Abrahan et al. 2020). However, studies that investigated the retrograde memory effect of novelty exposure yielded conflicting results and did not measure whether active anticipation of novelty enhances memory (Ballarini et al. 2013; Biel and Bunzeck 2019; Baumann et al. 2020). Consistent with findings that novelty anticipation—like reward motivation—recruits dopaminergic and memory-related brain regions (Lisman and Grace 2005; Bunzeck and Düzel 2006; Wittmann et al. 2007; Düzel et al. 2010), our findings provide the first direct behavioral evidence for the beneficial effect of anticipation of novelty on memory for incidental information.

Our results on novelty anticipation and incidental recollection are consistent with a recent study on reward anticipation showing that reward expectation enhanced memory for incidental objects that were presented early during reward anticipation potentially due to a reward-elicited phasic dopamine response (Stanek et al. 2019). The novelty-related memory enhancement observed in our study could be related to similar mechanisms due to phasic dopaminergic activity. Consistent with this idea, we found that the anticipation of novel environments enhanced recollection for incidental objects in proximity to the pathway starting point when the anticipation of novelty was elicited. Furthermore, our results are consistent with recent findings from our laboratory showing that incidental memory (for face images) was improved under high-curiosity compared with low-curiosity states only during an early phase of the anticipation period (Murphy et al. 2021). Therefore, our findings on the relationship between novelty anticipation and incidental memory together with reward- and curiosity-related effects on incidental memory suggest that potentially dopaminergic and hippocampal recruitment boost memory for incidental information that is in temporal proximity to the elicitation of dopaminergic activity.

Furthermore, the present findings contribute to the nascent literature of how curiosity impacts learning and memory. Prior studies on curiosity-related memory enhancements of incidental, unrelated material used (1) primarily trivia questions to elicit various levels of curiosity and (2) neutral face images as incidental material (Gruber et al. 2014; Galli et al. 2018; Stare et al. 2018; Fandakova and Gruber 2021; Murphy et al. 2021). Our findings that the overall level of curiosity (i.e., interindividual difference in curiosity) about novel rooms (instead of trivia answers) correlated with memory for incidental daily life objects (instead of faces) are in line with previous findings using the trivia paradigm on curiosity-related memory enhancements and the general notion of the positive effects of curiosity on learning and memory (Gruber and Ranganath 2019). Our findings on interindividual differences in curiosity for novel environments are also consistent with prior findings that interindividual differences in perceptual curiosity triggered by blurred images positively correlate with memory for these images (Jepma et al. 2012).

However, our finding that intraindividual differences in curiosity about novel environments negatively correlate with memory for incidental information encountered during anticipation was not predicted given earlier findings and theoretical accounts of curiosity and memory (e.g., Gruber and Ranganath 2019). Given that our current design had only three novel rooms, and thus only three trial-specific curiosity ratings per participant in our multilevel analysis, this negative intraindividual effect of curiosity on memory should be interpreted with some caution. However, the discrepancy between the current and prior findings on intraindividual effects on memory may be due to several differences between the VR paradigm used in the present study and the previously used trivia paradigm. One possibility for the different intraindividual curiosity effects on memory is the type of incidental stimuli. In previous studies using the trivia paradigm, faces are often used as incidental stimuli (e.g., Gruber et al. 2014; Fandakova and Gruber 2021; Murphy et al. 2021), whereas, here, objects were used as incidental stimuli. Compared with faces, objects are less salient and further studies would need to test whether the saliency of incidental stimuli might be essential in order for curiosity states to elicit positive spillover effects on incidental memory.

Potentially most importantly, the present VR paradigm had a series of six incidental items during the anticipation period rather than one item in previous experiments using the trivia paradigm (e.g., Gruber et al. 2014; Murphy et al. 2021). Depending on the amount of incidental information or the sequential position of the information, the effect of curiosity on memory could be different. For instance, a potential trade-off in cognitive resources due to the focus on navigating toward a novel room associated with high curiosity could mean that, with increased incidental information, less resources would be assigned to individual items. Furthermore, the anticipation period in our VR paradigm was longer (i.e., ∼26 sec) than in previous trivia paradigms (i.e., ∼10 sec) (Gruber et al. 2014; Murphy et al. 2021). Given that temporal proximity to the elicitation of curiosity is essential to trigger curiosity-related memory enhancements for incidental material (Murphy et al. 2021), most of the incidental objects in our current design might be encountered too late to benefit from an early dopaminergic response. In addition, more attention toward the novel outcome (i.e., room) at the end of the anticipation period could result in a negative impact on memory of incidental information, especially those objects close to the outcome.

Another possibility for the discrepancy in the intraindividual effect of curiosity is that the spatial environments of the present VR paradigm mainly elicit perceptual curiosity (i.e., curiosity for perceptual or sensory stimuli), whereas trivia questions trigger epistemic curiosity (i.e., curiosity for knowledge) (Berlyne 1954). It is interesting to note that one prior study showed that the elicitation of perceptual curiosity activated brain regions associated with cognitive conflict and arousal due to an aversive state (i.e., anterior insula and anterior cingulate cortex) (Jepma et al. 2012), whereas induction of epistemic curiosity by trivia questions activated brain regions associated with reward anticipation (e.g., SN/VTA and nucleus) (e.g., Kang et al. 2009; Gruber et al. 2014), Therefore, these two types of curiosity may involve different processes that could result in differential intraindividual effects on incidental memory for unrelated items through different neural pathways. Therefore, a fruitful avenue for future studies would be to systematically investigate how the effects of curiosity on incidental memory are modulated by different types of curiosity, the type of incidental information and the way incidental information is encountered during an anticipation phase.

In general, our exploratory analysis on how interindividual and intraindividual effects of curiosity about novel environments affect memory points toward a more nuanced effect of curiosity on incidental memory. The results suggest that curiosity might not always benefit memory, as suggested by previous studies using the trivia paradigm to test how epistemic curiosity affects memory. Future studies using the present design with more trials (i.e., rooms) would allow us to further investigate trial-specific curiosity for novel environments and its effects on memory, and to assess how the pattern of findings would differ with what has been observed using the trivia paradigm.

The present study asked the question of whether the beneficial effects of novelty and curiosity on memory reflect the same phenomenon or rely on distinct, potentially additive, processes. Our findings that interindividual differences in the level of curiosity for novel environments correlated with memory for incidental information support the latter. Specifically, our findings might be in line with the view that novelty is only a necessary requirement to elicit curiosity (Berlyne 1960; Gottlieb and Oudeyer 2018; Gruber and Ranganath 2019) and its positive effect of novelty on memory could be mediated through curiosity (Gruber and Ranganath 2019). Our findings on the interindividual relationship between curiosity and incidental memory might support the possibility that the general level of curiosity for novel information might be an important driver of novelty-related memory enhancements in previous studies. Here, participant-specific curiosity about novel virtual environments correlated positively with incidental memory. Anticipation of novel information evokes various levels of curiosity (accompanied with different levels of dopaminergic and hippocampal responses) and therefore different degrees of memory enhancement. To some extent, this view could explain the conflicting results in prior studies that found retrograde novelty-related memory enhancements (Ballarini et al. 2013) while others did not (Biel and Bunzeck 2019). We speculate that when novel stimuli generally elicit higher levels of curiosity, participants who show higher overall curiosity in novel stimuli would observe greater “novelty”-related memory enhancements. In contrast, when novel stimuli are unable to elicit high curiosity in some participants, the relatively small or lacking recruitment of dopaminergic and hippocampal responses could be insufficient to produce memory enhancements.

At the intraindividual level, however, the negative correlation between the level of curiosity for the individual room and memory for the associated incidental objects suggests that curiosity for novel environments could also have a detrimental effect on memory for incidental information. Given the low trial numbers of curiosity ratings, we are cautious about drawing strong conclusions from this negative relationship between intraindividual curiosity and incidental memory. Nevertheless, this finding might suggest that variations in trial-specific curiosity and novelty might affect memory in opposing manners, with worse incidental memory during curiosity states when curiosity for a specific novel environment is particularly high resulting in increased competition of cognitive resources between curiosity target and incidental information encountered during curiosity states. To further understand how novelty and intraindividual curiosity jointly affect memory for novel and incidental information during novelty anticipation, future studies investigating novelty-related memory effects would benefit from adding measures of curiosity.

Both the novelty-related memory enhancement and the inter/intraindividual effects of curiosity on memory were observed across the immediate and delayed memory tests. Despite the theoretical idea that novelty might primarily improve memory via consolidation (i.e., by enhancing long-term potentiation) (Lisman and Grace 2005), several studies in humans have robustly shown an immediate positive effect of novelty exposure on memory for incidental information (e.g., Fenker et al. 2008; Schomaker et al. 2014; Schomaker and Wittmann 2021) and also of high-curiosity states on memory for incidental face images (e.g., Gruber et al. 2014; Galli et al. 2018; Stare et al. 2018; Murphy et al. 2021). Therefore, the findings further suggest that cognitive and/or neuromodulatory effects on memory via novelty, reward or curiosity might also be driven by processes during initial encoding rather than primarily consolidation-related processes (c.f., Shohamy and Adcock 2010).

Finally, our findings bridge the learning-promoting and motivational aspects of novelty, suggesting that novelty and curiosity may be two distinct but closely related processes in affecting memory. Furthermore, the current study demonstrates the usefulness of our developed VR paradigm for future studies to investigate curiosity and its effects on memory and exploration in novel environments.

## Supplementary Material

Supplemental Material
