# Supplementary material for: Anticipation of novel environments enhances memory for incidental information
Source: Learn Mem. 2021 Aug;28(8):254–9. doi: 10.1101/lm.053392.121 (PMC8284314; doi:10.1101/lm.053392.121)
Supplement: Supplemental Material [file supp_28_8_254__DC1.html]

Supplemental Material 

# Anticipation of novel environments enhances memory for incidental information

## Supplemental Material

- SupplementaryInformation.docx
