# Supplementary material for: Anticipation of novel environments enhances memory for incidental information
Source: Learn Mem. 2021 Aug;28(8):254–9. doi: 10.1101/lm.053392.121 (PMC8284314; doi:10.1101/lm.053392.121)
Supplement: Supplemental Material [file supp_28.8.254_SupplementaryInformation_.docx]

Table of Contents

[Supplementary Method 2](#_Toc70769090)

[Participants 2](#_Toc70769091)

[Stimuli and apparatus 2](#_Toc70769092)

[Design and Procedure 3](#_Toc70769093)

[Familiarisation Phase 3](#_Toc70769094)

[Encoding Phase 4](#_Toc70769095)

[Memory Tests 6](#_Toc70769096)

[Data Analysis 6](#_Toc70769097)

[Supplementary Results 8](#_Toc70769098)

[Novelty-related memory enhancement as a function of distance 8](#_Toc70769099)

[Curiosity ratings between novel and familiarized rooms 10](#_Toc70769100)

[Effect of novelty anticipation on familiarity-based recognition and source memory accuracy for incidental objects 11](#_Toc70769101)

[Memory performance for objects inside novel and familiarised rooms 12](#_Toc70769102)

[Supplemental References 15](#_Toc70769103)

# Supplementary Method

## Participants

All 82 participants gave their written informed consent for the procedures and received course credits or monetary compensation for their participation. The study was approved by the ethical committee of the School of Psychology at Cardiff University, Wales, UK, and performed in accordance with the ethical standards laid down in the Declaration of Helsinki.

## Stimuli and apparatus

The virtual environment consisted of an outdoor scene, which remained the same for all trials, and multiple indoor scenes (i.e., the virtual rooms) which varied across trials. As illustrated in Figure 1A, the outdoor scene contained a pier with a zigzag-shaped pathway connecting the pier and a virtual room. In each trial, the participants would start from the pier and navigate through the pathway towards a virtual room that participants subsequently explored. The virtual world including the outdoor scene was created in Unity 3D (version 2018.2.1f1, Unity Technologies).

Six rooms were created for exploration: *Bedroom*, *Classroom*, *Library*, *Gym*, *Living Room* and *Storage Room*. In each trial, the participants would visit one of the rooms. The type of room was labelled on the front wall of the room so that participants could read from the start of the trial (i.e. standing at the pier) which room they were going to visit. The rooms were created using SketchUp ([www.sketchup.com](http://www.sketchup.com)) and then imported into the virtual world in Unity 3D. The six rooms were separated into two groups. One group contained *Bedroom*, *Classroom* and *Gym*, and the other group contained *Library*, *Living Room* and *Storage Room.* The groups were counterbalanced between participants across the familiarised and novel room conditions in order not to induce any material effects.

A total of 108 3D models of daily objects were downloaded from online 3D stores ([www.sketchfab.com](http://www.sketchfab.com/) and [www.turbosquid.com](http://www.turbosquid.com/)) (see Figure 1C for object examples). These 3D models were selected based on their resemblance to their real-life counterpart and size. Models of large objects such as airplanes were not included as it would be unrealistic to place them in real size on the pathway. The 3D models were selected from a wide range of object categories common in daily life, including food, electronic devices, groceries and tools. Among these object models, 72 models served as encoding stimuli. Half of the objects were presented during the anticipation phase as incidental material and the other half were presented inside the rooms. These object models were counterbalanced across outside and inside the rooms and randomly assigned across trials so that each room would not be tied to a particular type of object. The remaining 36 object models served as lures during the memory tests (see Figure 1D for a lure example).

The experiment was run in Unity game engine on a desktop PC. The visual stimuli were presented on an LCD monitor (1920 × 1080 pixels; 60 Hz refresh rate). Participants were seated at a viewing distance of about 75 cm. While moving around the virtual world, participants could use the mouse to steer their direction and the keyboard to move forward (by pressing “W”) or backward (“S”). To increase the sense of presence and agency, participants could hear footstep sounds while moving around. Sounds were presented through headphones. Note that participants were unable to move sideways in a crablike way along the pathway. The speed of movement was fixed to be 2 eye-heights per second resembling a brisk walking speed in the virtual world and ensured that participants spent roughly equal time on the pathway.

## Design and Procedure

All participants went through three phases: the familiarisation phase, encoding phase and memory test phase.

### Familiarisation Phase

During the familiarisation phase (Figure 1B), participants explored all three rooms from one of the two groups, counterbalanced between participants. This phase allowed the participants to familiarise themselves with navigating in the virtual world and to learn about the interior and layout of each room in the familiarised room condition.

In each familiarisation trial, the participants explored one room. They started from the pier and then moved through the pathway to the front of the room. The door of the room was closed, and the participants needed to press “E” to open it. The duration of opening the door was 5 seconds. Once the door was open, the participants could enter the room and freely explore inside. They remained in the room for one minute and were encouraged to explore as much as possible. After one minute, the trial was terminated, and the participants were teleported back to the pier and the next trial started. After exploring all three rooms of the familiarised condition, participants were asked to draw a ground plan for each room they just explored. If they were unable to draw at least two-thirds of the items inside or the layout of the room accurately, they would be asked to do the familiarisation phase again to ensure that they were fully familiar with the rooms. None of the participants included in the final analyses did the familiarisation phase more than once. During the familiarisation phase, no incidental objects that would later be tested were presented on the pathway or inside the rooms.

### Encoding Phase

During the encoding phase, participants visited all six rooms, which were evenly grouped into three blocks, with each block containing one familiarised room and one novel room. At the beginning of each block, participants were presented pictures of the two rooms so that they knew which rooms they would be visiting in a given block. The basic procedure in each encoding trial was the same as in a familiarisation trial, with some important additions: At the start, participants’ view was frozen, and they were standing at the pier and seeing the particular room label on the front wall of the room. A question display was overlaid, asking how curious they felt about the room. They were instructed to rate their curiosity on a Likert scale from 1 (“*Not at all curious*”) to 7 (“*Extremely curious*”) (Figure 1C) by inputting an integer number using the keyboard. Participants were encouraged to spread their ratings on the scale as much as possible and not to stick to the two extremes or the middle range of the scale. As shown by the distribution of curiosity ratings in Figure S1 in Supplementary Results, almost all the participants followed this instruction. After the participants submitted the rating, the question display disappeared, and they were able to start walking towards the room. As in the familiarisation phase, participants started from the pier and moved along the zigzag-shaped pathway leading up to the room. On the pathway, they would see six objects separately allocated in the corners, e. g., the toaster and camera to the familiarised room and the moka express and grapes to the novel room in Figure 1C. As each object was occluded by the pathway fence, participants could not see it until they walked on the part of the pathway facing the corner where the object was placed. An invisible restriction was imposed so that participants were able to move at the predefined speed (i.e. 2 eye-heights per second) only when facing the object ahead. Otherwise, their movement would become slow and jittery. By doing this, the view duration was made comparable across objects, trials and participants. Participants were told that the objects were not relevant, and that no particular action on the objects was required. They were instructed to keep walking and not to stop for the objects. When they passed the object, the object disappeared with a “cling” sound to further support encoding of the incidental objects in addition to viewing the objects while walking.

In addition to incidental objects outside the rooms, participants were given an encoding task for objects they would see inside the rooms. This task was initially included for the purpose of examining memory enhancement for items encoded within novel compared to familiarised environments. When participants entered the room, they were instructed to find and collect six objects that were separately hidden inside six wooden boxes that were randomly allocated inside the room. After picking up all six objects, participants could leave the room by pressing “B” and end the trial. A 4-minute time limit was imposed for staying in the room to prevent overexposure. At a later point of the study, we realised that the search task inside the rooms interfered with the novel/familiarised room manipulation. Consequently, we did not find that objects within novel rooms showed better memory compared to objects in familiarised rooms. For the sake of completeness, we included memory performance of objects inside the rooms in the Supplementary Results (see Supplemental Figure S4 and Table 2).

### Memory Tests

Surprise memory tests took place either immediately or 24 hours after the encoding phase (Figure 1D), separately for the objects on the pathway leading to the rooms and objects inside the rooms. In each memory test, the 36 objects that had been seen in the encoding phase and 18 lures were presented on the screen one at a time in a randomised and intermixed sequence. For each presented object, participants indicated whether it was “remembered”, “familiar” or “new” by pressing the corresponding key on the keyboard (1 – “remembered”, 2 – “familiar”, and 3 – “new”). After responding to the object image, participants were shown four pictures: three pictures of rooms and one picture with a question mark. Participants were asked to choose the picture of the room that they thought was associated with the object (i.e., leading to or inside which room the object was seen). If they thought the object was new, they were instructed to choose the picture displaying a question mark. The memory tests were self-paced and there was a short break between the two memory tests. As memory for the object-room associations was not above chance for incidental objects, we did not include the performance in the final analysis.

### Data Analysis

To explore the effects of curiosity on memory at the inter- but also intra-individual levels, we used the “nlme” package in R (Pinheiro et al., 2007) to construct a multilevel linear regression with participant as the random effect, curiosity rating for each novel room as the level-1 predictor, and individual average curiosity rating across three novel rooms and memory test (*immediate* and *delayed*) as the level-2 predictor. In this way, the parameters associated with individual average curiosity rating would show curiosity effect at the inter-individual level, and the parameters associated with curiosity rating *per* room would show curiosity effect at the intra-individual level. See the model below,

$Accuracy_{ij}= \gamma_{00}+\gamma_{01}\left( AverageCuriosity_{j} \right)+\gamma_{02}\left( MemoryTest_{j} \right)+\gamma_{10}\left( Curiosity_{ij} \right)+[residuals]$,

where $Accuracy_{ij}$ refers to the $j$th participant’s recollection accuracy for the incidental objects leading to the $i$th room, $AverageCuriosity_{j}$ refers to the $j$th participant’s average curiosity rating across the three novel rooms, $MemoryTest_{j}$ refers to the memory test group (*immediate* or *delayed*) that the $j$th participant was in, $Curoisity_{ij}$ refers to the $j$th participant’s curiosity rating for the $i$th room, $residuals$ the general residual variance.

Individual average curiosity ratings were grand-mean centred, and curiosity ratings for novel rooms were participant-mean centred. This would remove all between-participant variation in the within-participant-level predictors (Enders & Tofighi, 2007)

Familiarity-based recognition accuracy was calculated by conditionalizing on the opportunity to make a “familiar” response when a “remembered” response was not made (Libby et al., 2013), following the equations below:

$$Familiarity_{old items}=\frac{Familiarit{y \left( "familiar" response \right)}_{\text{old items}}}{1-Remembered \left( "remembered" response \right)_{old items}}$$

$$Familiarity_{new items}=\frac{Familiarit{y \left( "familiar" response \right)}_{\text{new items}}}{1-Remembered \left( "remembered" response \right)_{new items}}$$

Then, familiarity-based recognition accuracy is estimated as:

$$Familiarity=Familiarity_{old items}- Familiarity_{new items}$$

Source memory performance was estimated by correct hit rate. As there were four options (i.e., three pictures of room and one picture of question mark), the chance for correct hit rate is 25%.

# Supplementary Results

## Novelty-related memory enhancement as a function of distance

Novelty-related memory enhancement was calculated as the difference between the novel rooms and familiarised rooms (novel – familiarised) for each object position (from the 1^st^ to 6^th^ object position relative to the starting point) for each individual participant. Then, a linear multilevel analysis was performed on the difference score, with object position as the level-1 predictor and participant as the random effect. Memory test (immediate and delayed memory test group) and its interaction with object position was subsequently added to the model as level-2 predictors, but this did not substantially improve the model fit (both *p*s > 0.1). Therefore, we did not include memory test and its interaction in the final model.

The analysis revealed a significant effect of novelty anticipation on the intercept of the final model (b = 8.56, *p* < 0.01), indicating that recollection accuracy was significantly higher for the object that is the closest to the starting point (i.e., the early part of the anticipation period) within the novel compared to the familiarised condition. This novelty-related memory enhancement has an overall trend of decreasing over distance (b = -1.69, *p* = 0.069). In addition, exploratory separate t-tests showed a significant novelty-related memory enhancement for the 1^st^ (t = 2.49, *p* = 0.015) and 2^nd^ object (t = 2.05, *p* = 0.044), but not for objects at the later positions (all *p*s > 0.1). These results suggest that the novelty-related memory enhancement might be mostly driven by processes during the early phase of anticipation.

***Figure S1.*** *Mean difference in recollection accuracy for incidental objects encountered on the pathway leading to novel compared to familiarised rooms (novel – familiarised) at each object location along the pathway (from the starting point to the room). The red line illustrates the fixed effect of object distance predicted by the multilevel analysis. Error bars represent 95% confidence intervals.*

## Curiosity ratings between novel and familiarized rooms

***Figure S2.*** *Average curiosity ratings for the familiarized rooms (green) and novel rooms (red). Histograms (shaded areas) shows the distribution of the individual average curiosity ratings in the condition. Dots represent the average curiosity rating of the participant for the condition. Boxplots below the histograms illustrate the median, the 25^th^ and 75^th^ percentiles of the individual average curiosity ratings for the condition.*

## Effect of novelty anticipation on familiarity-based recognition and source memory accuracy for incidental objects

A 2 (room novelty: novel vs. familiarised) × 2 (memory test: immediate vs. delayed) mixed ANOVA was computed on familiarity-based recognition. The analysis did not reveal any significant effect of room novelty (novel vs. familiarised) (F_1, 77_ = 0.70, *p* = 0.41) or memory test (immediate vs. delayed) (F_1, 77_ = 0.26, *p* = 0.61), or any interaction (F_1, 77_ = 0.72, *p* = 0.40). Given that the study was aiming to test whether curiosity could further correlate with novelty-related memory enhancement, we did not proceed to examine the effect of curiosity on familiarity-based recognition any further.

The mean hit rate of source memory for each condition was shown in Table S1. In none of the conditions, the source memory hit rate was higher than the chance rate (all *p*s > 0.05). Due to this floor effect, we did not proceed with analysis on source recognition memory any further.

***Figure S3.*** *Average familiarity-based recognition accuracy for novel rooms (dark purple) and familiarized rooms (light purple). Error bars indicate 95% confidence intervals.*

***Table S1.*** *Average accuracy for familiarity-based recognition and source memory.*

|  | **Familiarity-based Recognition Accuracy** | **Source Memory  Hit Rate** |
| --- | --- | --- |
| **Immediate memory test group** |  |  |
| Novel rooms | M = 14.43%, SE = 3.64%, 95% CI [7.06%, 21.8%] | M = 25%, SE = 2.11%, 95% CI [20.72%, 29.28%] |
| Familiarised rooms | M = 14.43%, SE = 2.96%, 95% CI [8.43%, 20.42%] | M = 19.58%, SE = 1.8%, 95% CI [15.94%, 23.23%] |
| **Delayed memory test group** |  |  |
| Novel rooms | M = 10.44%, SE = 2.94%, 95% CI [4.48%, 16.41%] | M = 20.3%, SE = 1.66%, 95% CI [16.94%, 23.66%] |
| Familiarised rooms | M = 14.86%, SE = 2.66%, 95% CI [9.48%, 20.24%] | M = 20.41%, SE = 1.73%, 95% CI [16.9%, 23.92%] |

## Memory performance for objects inside novel and familiarised rooms

Recollection accuracy for objects encoded inside the rooms was significantly higher in the immediate memory test group than in the delayed memory test group (F_1, 77_ = 12.46, *p* < 0.01, $\eta_{p}^{2}$ = 0.14), as shown in Figure S4A. There was no main effect of room novelty (F_1, 77_ = 2.77, *p* = 0.10), but a significant interaction between room novelty and memory test (F_1, 77_ = 6.60, *p* = 0.012, $\eta_{p}^{2}$ = 0.079). Unexpectedly, separate paired t-tests showed that, for the immediate memory test group, recollection accuracy was significantly higher for familiarised rooms than novel rooms (t_39_ = 3.98, *p* < 0.01). No significant difference was found in recollection accuracy between novel and familiarised rooms in the delayed memory test group (t_40_ = 0.55, *p* = 0.59).

For familiarity-based recognition, no main effect of memory test (F_1, 74_ = 2.89, *p* = 0.093) or room novelty (F_1, 74_ = 0.57, *p* = 0.45), or interaction (F_1, 74_ = 0.076, *p* = 0.78) was found.

Consistent with our approach for source memory for incidental objects, we first checked whether source memory for objects inside the rooms was above chance for each condition. Separate t-tests showed that in all conditions, source recognition performance was higher than chance (i.e., 25%) (all *p*s < 0.01). Therefore, we continued to examine the effect of novelty. The 2 (room novelty: novel vs. familiar) × 2 (memory test: immediate vs. delayed) mixed ANOVA showed that there was a main effect of memory test (F_1, 77_ = 4.89, *p* = 0.03, $\eta_{p}^{2}$ = 0.06) with accuracy was significantly higher for the immediate than delayed memory test group. No main effect of room novelty (F_1, 77_ = 0.84, *p* = 0.36) or interaction (F_1, 77_ = 1.78, *p* = 0.19) was found.

Since we did not find any novelty-related positive modulation on memory for objects inside the rooms, we did not follow up on how curiosity might correlate with memory for objects in novel rooms.

***Figure S4.*** *Mean recollection accuracy (****A****), mean familiarity-based recognition accuracy (****B****) and mean source memory hit rate (****C****) for objects encoded inside the rooms. The data are averaged across the participants for the immediate (N = 40) and delayed (N = 39) memory tests separately. In (****B****), data of three participants from the immediate memory test group were excluded because they had 100% hit rate for correct “remembered” response and made no “familiar” response. The grey dashed line at 25% in (****C****) indicates the chance rate for source memory hit rate. Error bars in all panels depict 95% confidence interval.*

***Table S2.*** *Average accuracy for recollection, familiarity-based recognition and source recognition performance for objects collected inside the rooms.*

|  | **Recollection Accuracy** | **Familiarity-based Recognition Accuracy** | **Source Memory  Hit Rate** |
| --- | --- | --- | --- |
| **Immediate  memory test group** | |  |  |
| Novel rooms | M = 65.19%, SE = 3.17%, 95% CI [58.78%, 71.6%] | M = 33.87%, SE = 4.8%, 95% CI [24.15%, 43.6%] | M = 37.19%, SE = 2.88%,  95% CI [31.36%, 43.02%] |
| Familiar rooms | M = 72.21%, SE = 3.02%, 95% CI [66.1%, 78.31%] | M = 30.26%, SE = 4.94%, 95% CI [20.24%, 40.28%] | M = 37.96%, SE = 2.55%,  95% CI [32.81%, 43.11%] |
| **Delayed  memory test group** | |  |  |
| Novel rooms | M = 54.87%, SE = 3.41%, 95% CI [47.97%, 61.77%] | M = 42.86%, SE = 5.05%, 95% CI [32.63%, 53.08%] | M = 33.41%, SE = 2.19%, 95% CI [28.97%, 37.85%] |
| Familiar rooms | M = 53.3%, SE = 3.01%, 95% CI [47.2%, 59.41%] | M = 41.17%, SE = 4.45%, 95% CI [32.16%, 50.17%] | M = 29.05%, SE = 2.03%, 95% CI [24.93%, 33.16%] |

#

# Supplemental References

Enders, C. K., & Tofighi, D. (2007). Centering Predictor Variables in Cross-Sectional Multilevel Models: A New Look at an Old Issue. *Psychological Methods*, *12*(2), 121–138. https://doi.org/10.1037/1082-989x.12.2.121

Libby, L. A., Yonelinas, A. P., Ranganath, C., & Ragland, J. D. (2013). Recollection and Familiarity in Schizophrenia: A Quantitative Review. *Biological Psychiatry*, *73*(10), 944–950. https://doi.org/10.1016/j.biopsych.2012.10.027

Pinheiro, J., Bates, D., DebRoy, S., Sarkar, D., & Team, R. C. (2007). Linear and nonlinear mixed effects models. *R package version*, *3*(57), 1–89.
